# Efficient analysis of stochastic gene dynamics in the non-adiabatic regime using piecewise deterministic Markov processes

**DOI:** 10.1098/rsif.2017.0804

**Published:** 2018-01-31

**Authors:** Yen Ting Lin, Nicolas E. Buchler

**Affiliations:** 1 Theoretical Division and Center for Nonlinear Studies, Los Alamos National Laboratory, Los Alamos, NM 87545, USA; 2 School of Physics and Astronomy, University of Manchester, Manchester M13 9PL, UK; 3 Department of Physics, Duke University, Durham, NC 27708, USA; 4 Department of Biology, Duke University, Durham, NC 27708, USA; 5 Center for Genomic and Computational Biology, Durham, NC 27710, USA

**Keywords:** mathematical model, gene expression, circadian rhythm, intrinsic noise, stochastic cycles

## Abstract

Single-cell experiments show that gene expression is stochastic and bursty, a feature that can emerge from slow switching between promoter states with different activities. In addition to slow chromatin and/or DNA looping dynamics, one source of long-lived promoter states is the slow binding and unbinding kinetics of transcription factors to promoters, i.e. the non-adiabatic binding regime. Here, we introduce a simple analytical framework, known as a piecewise deterministic Markov process (PDMP), that accurately describes the stochastic dynamics of gene expression in the non-adiabatic regime. We illustrate the utility of the PDMP on a non-trivial dynamical system by analysing the properties of a titration-based oscillator in the non-adiabatic limit. We first show how to transform the underlying chemical master equation into a PDMP where the slow transitions between promoter states are stochastic, but whose rates depend upon the faster deterministic dynamics of the transcription factors regulated by these promoters. We show that the PDMP accurately describes the observed periods of stochastic cycles in activator and repressor-based titration oscillators. We then generalize our PDMP analysis to more complicated versions of titration-based oscillators to explain how multiple binding sites lengthen the period and improve coherence. Last, we show how noise-induced oscillation previously observed in a titration-based oscillator arises from non-adiabatic and discrete binding events at the promoter site.

## 1. Introduction

Gene expression is fundamentally a stochastic biochemical process that arises from thermal fluctuations. An important source of stochastic noise comes from the discrete and random binding and unbinding events between the regulating transcription factors (TFs) and the promoter sites of the regulated genes. Conventionally, these DNA binding and unbinding events are thought to be fast compared with the downstream processes of transcription, translation and degradation [1]. This separation of timescales leads to an approximation, known as a quasi-steady state or adiabatic approximation, where the mean transcription rate simplifies to a function of the concentrations and protein–DNA dissociation constants at the promoter [2,3]. The adiabatic approximation is commonly used to reduce the number of dynamical variables (e.g. promoter states) in gene regulatory networks. However, it is also a bold assumption because experiments [4–7] show that promoter dynamics (e.g. the binding and unbinding events of TFs) can take place at a comparable, or even slower, timescale than the downstream processes of gene expression. This observation has motivated theoretical studies into the effects of slow or non-adiabatic binding on gene regulatory networks. There is a consensus that non-adiabatic binding results in bursty production of transcripts [8,9], broadened distributions of gene expression [10–12] and bi- or multi-stabilities that reflect the discrete, underlying promoter states [11–13].

Many of these studies focused on the system properties at stationarity and mostly ignored the effects of non-adiabatic binding on the non-equilibrium dynamics of gene regulatory networks. In this article, we address the following questions: what are the *dynamical* consequences of non-adiabatic binding? What kind of modelling framework accurately describes the non-stationary dynamics of gene regulatory networks in the non-adiabatic regime? To answer these questions, we use a model of titration-based clocks to illustrate the effects of non-adiabatic binding on dynamics (e.g. oscillation) and to show how an analytical framework, known as a piecewise deterministic Markov process (PDMP), accurately describes the stochastic dynamics of the full model in the non-adiabatic regime. The PDMP is an efficient method of analysis that is valid regardless of the mechanism (e.g. slow chromatin, DNA looping, unbinding kinetics) responsible for slow-switching promoters.

This article is organized as follows. In §2.1, we introduce two idealized models of titration-based circuits commonly found in circadian clocks and immune signalling. We prove in §2.2 that limit cycles are impossible in the fast-binding (adiabatic) limit. In §2.3, we simulate the full chemical master equation (CME) to demonstrate that the titration-based circuits exhibit stochastic cycles in the slow-binding (non-adiabatic) limit. We then transform the CME into a PDMP where transitions between discrete promoter states are stochastic but the rates depend upon the faster deterministic dynamics of the transcription factor concentrations regulated by these promoters (§2.4). The PDMP makes no assumptions regarding the timescales of promoter switching and is valid for both slow or fast switching. It is an exact formulation of the CME in the thermodynamic limit for systems where protein numbers are large. The thermodynamic limit and, hence, PDMP analysis, is well suited for stochastic gene dynamics in eukaryotic cells where cell sizes and the number of regulatory proteins can be large. We show that the PDMP framework accurately describes the observed periods and coherence of stochastic cycles in the non-adiabatic regime. We also demonstrate that the PDMP can be readily applied to more detailed and mechanistic models in §3. We conclude in §4 by discussing our results and PDMP analysis in the context of previous work on non-adiabatic binding and oscillation in gene networks.

## 2. Mathematical framework

We begin by introducing two idealized models of titration-based gene regulatory networks commonly found in biological oscillators. These models are ‘idealized’ in the sense that transcription and translation are lumped into a single-stage of ‘production’, and the intermediate mRNA populations are not explicitly modelled. We further simplify the cis-regulatory architecture of each promoter to the fewest number of possible binding states. The purpose of these idealized models is to illustrate how PDMP analysis can be used to understand the origin and properties of the stochastic cycles that emerge in the non-adiabatic regime. We will relax some of these assumptions in later sections.

### 2.1. Idealized models

**Figure 1.**
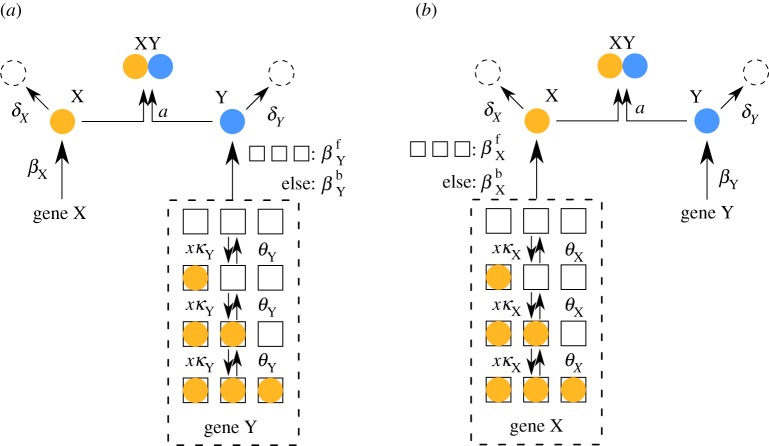
Schematic diagrams of the idealized (*a*) activator-titration circuit (ATC) and (*b*) repressor-titration circuit (RTC). Protein *X* is a transcription factor and *Y* is an inhibitor that can irreversibly associate with *X* to form an inactive complex. In the ATC, *X* is an activator that can sequentially bind multiple DNA sites in the promoter of gene *Y* and increase transcription of the inhibitor. In the RTC, *X* is a repressor that can bind its own promoter and repress transcription. (Online version in colour.)

Both idealized models consist of two genes, which produce two kinds of regulatory proteins *X* (a TF) and *Y* (an inhibitor that titrates *X* into an inactive complex; figure 1). Our first model is called the activator-titration circuit (ATC) because protein *X* is a transcriptional activator [14,15]. In this model, *X* increases the production rate of inhibitor *Y* by binding to cis-regulatory binding sites in the promoter of gene Y with an association rate *κ*_Y_. There are a total of 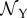 cis-regulatory binding sites in the promoter of gene Y and we assume that binding of X is sequential, such that there are a total of 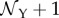 promoter states. Bound *X* dissociates sequentially from each binding site with a rate *θ*_Y_. The production rate of gene Y depends nonlinearly on the number of *X* bound to the promoter because the production rate is *β*^b^_Y_ (bound) when any of the binding sites are occupied; otherwise, the production rate is *β*^f^_Y_ (free). We note that *β*^b^_Y_ > *β*^f^_Y_ because *X* is an activator. Gene X is unregulated and, thus, activator *X* is constitutively produced at a constant rate *β*_X_. Last, inhibitor *Y* inhibits the activity of TF *X* by titration, where one *Y* molecule irreversibly binds to one *X* molecule with a bimolecular rate of association (*α*) and forms a non-functional heterodimer. The idealized ATC can be modelled by the following elementary reactions:
2.1
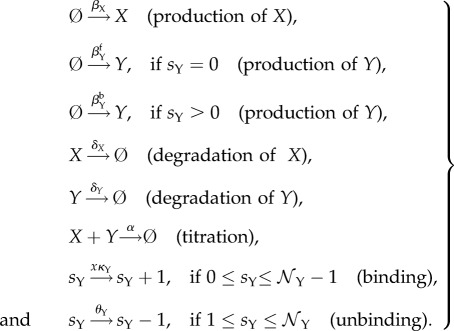
Here, 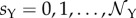 identifies the promoter state by its number of bound *X*. A schematic diagram of the ATC can be found in figure 1*a*. All the elementary rate constants are defined in the sense of mass action kinetics, and *x* denotes the concentration of TF *X* in the thermodynamic limit. In the stochastic models that consider discrete molecules (detailed in §2.3), the rates have to be properly scaled by *Ω*, which is a parameter that quantifies the system size and is related to cell volume. The scaling relationship between the mass-action rate constants and the stochastic model rates can be found in appendix A.

The second model is called a repressor-titration circuit (RTC) because *X* is a transcriptional repressor [15] (figure 1*b*). This model differs from the ATC in two ways. First, the inhibitor *Y* is now constitutively expressed at a constant rate *β*_Y_. Secondly, *X* negatively auto-regulates itself where *X* decreases its own production rate by binding to cis-regulatory binding sites in the promoter of gene X with an association rate *κ*_X_. There are a total of 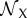 cis-regulatory binding sites in the promoter of gene X and we assume that binding of X is sequential, such that there are a total of 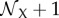 promoter states. Bound *X* dissociates sequentially from each binding sites with a rate *θ*_X_. The production rate of gene X depends nonlinearly on the number of *X* bound to the promoters, where the production rate of *X* is *β*^b^_X_ (bound) when any of the binding sites are occupied; otherwise, the production rate is *β*^f^_X_ (free). We note that *β*^f^_X_ > *β*^b^_X_ because *X* is a repressor. The rest of the process and parameters are similarly defined as in the ATC. The idealized RTC can be modelled by the following elementary reactions:
2.2
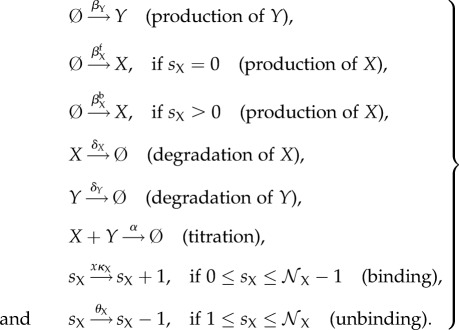
Similarly, 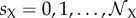 identifies the promoter state by its number of bound *X*.

### 2.2. No limit cycle in the adiabatic limit

The aim of this section is to show that mass action kinetics describing the ATC and RTC in the fast-switching (adiabatic) limit do not allow deterministic limit cycles. Below, we generically use Z = X or Y as the gene index and *Z* = *X* or *Y* as the protein index. The discrete switching events between the bound TF at the promoter sites, 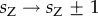, are a random birth-and-death process [16,17] where the birth rate *κ*_Z_ depends on the concentration (*x*) of the transcription factor (TF) *X*. In the fast-switching (adiabatic) limit, formally expressed as 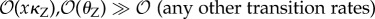, the variable *x* is a slow variable and is treated as approximately constant. In this limit, the birth and death rates are approximately constant, and the quasi-stationary distribution (QSD) of *s*_Z_ is obtained using detailed balance of this one-dimensional birth–death process [16]:
2.3
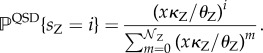
The effective production rate of the regulated gene, *β*^eff^_Z_, can be derived using the QSD (2.3):
2.4
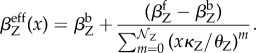


In the thermodynamic limit, we denote the concentrations of *X* and *Y* by *x* and *y* respectively, and the resulting mass action kinetics of the ATC and RTC are described by the following deterministic differential equations:
2.5a
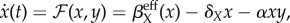
and
2.5b
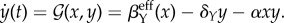
For simplicity, we unified the expressions for the idealized ATC and RTC where *β*^eff^_X_(*x*):= constant *β*_X_ in the ATC and *β*^eff^_Y_(*x*):= constant *β*_Y_ in the RTC. Equations (2.5) constitute a two-dimensional dynamical system. The Bendixson criterion [18] states that limit cycles do not exist when the trace of the Jacobian, 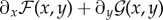, does not change sign on a simply connected domain. On the biologically relevant domain *x* ≥ 0 and *y* ≥ 0,
2.6
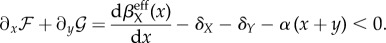
The trace of the ATC is always negative because d*β*^eff^_X_(*x*)/d*x* = 0. The trace of the RTC is also always negative because d*β*^eff^_X_(*x*)/d*x* < 0. Thus, there are no deterministic limit cycles for the idealized ATC and RTC in the adiabatic limit. If we were to modify the ATC such that the activator *X* also stimulates its own production (i.e. positive feedback), then d*β*^eff^_X_(*x*)/d*x* > 0 and it would be possible to have limit cycles in the adiabatic limit.

### 2.3. Stochastic cycles in the non-adiabatic regime

We first develop a full stochastic model that describes the dynamics where the population of molecules and the number of bound promoter sites are all discrete. We will then use this model to show the emergence of stochastic cycles with well-defined periods in the non-adiabatic regime. The state of the model is determined by (i) the population of *X*, *N*_*X*_, (ii) the population of *Y* , *N*_*Y*_, (iii) the bound promoter state of gene X, *s*_X_ and (iv) the bound promoter state of gene Y, *s*_Y_. The probability of having *N*_*X*_ = *i*, *N*_*Y*_ = *j*, *s*_X_ = *k*, and *s*_Y_ = *l* at time *t* is given by *P*_*i*,*j*,*k*,*l*_(*t*). The discrete-state stochastic process of the idealized model is described by the CME [16,17]:
2.7
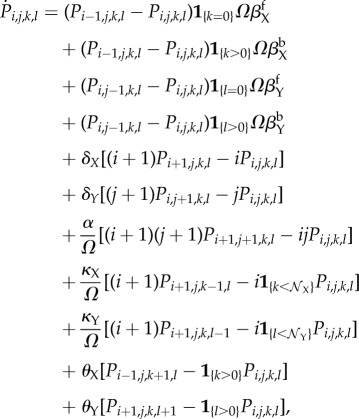
where we have suppressed writing the *t*-dependence of *P*_*i*,*j*,*k*,*l*_ for brevity. The boundary conditions *P*_*i*,*j*,*k*,*l*_ = 0 when *i* < 0, *j* < 0, *k* < 0, *l* < 0, 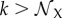 or 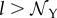 are imposed. We unified the model descriptions of ATC and RTC; for ATC, 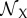, *κ*_*X*_, *θ*_*X*_: = 0 and *β*^b^_X_ = *β*^f^_X_ = constant *β*_X_; similarly, for RTC, 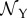,*κ*_*Y*_, *θ*_*Y*_: = 0 and *β*^b^_Y_ = *β*^f^_Y_ = constant *β*_Y_. **1**_{condition}_ is the characteristic function: it is equal to 1 when the condition is true; otherwise 0. The different rates and the protein population scale as a function of system size *Ω*, as outlined in appendix A [11,16]. The concentrations *x* and *y* in previous sections are interpreted as the normalized population density *N*_*X*_/*Ω* and *N*_*Y*_/*Ω*, where *N*_*X*_ and *N*_*Y*_ are the discrete populations of regulatory proteins *X* and *Y* .

We refer to the model (2.7) as the full CME. Standard continuous time Markov chain simulations were constructed to generate exact sample paths of the full CME of the ATC and RTC [19,20]. We chose two sets of parameters listed in table 1, where the ATC and RTC promoters have a single binding site (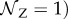 and the only source of nonlinearity is the titration of *X* by *Y* . We chose this parameter set because it is simple and it illustrates the fundamental ingredients of stochastic cycling in the non-adiabatic regime. We will consider more complicated cis-regulatory promoters in later sections. For each parameter set, we introduce a scaling factor *λ*, such that the binding and unbinding rates are, respectively, parametrized by 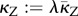 and 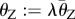. We fixed 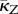 and 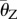 and systematically change the value of *λ* in order to examine the dynamics of the same model in both the adiabatic and non-adiabatic regime.

**Table 1.** Descriptions and values of the idealized model parameters.

parameter	description	ATC	RTC	order of reaction
*Ω*	characteristic system size	10^3^	10^3^	n.a.
*λ*	scaling factor of the binding rate *κ*_Z_ and unbinding rate *θ*_Z_, Z ∈ {X, Y}	(1, 10^3^)	(1, 10^3^)	n.a.
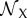	number of binding sites on gene X	0	(1, 3)	n.a.
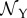	number of binding sites on gene Y	(1, 3)	0	n.a.
*β*^f^_X_	basal production rate of gene X when the number of bound 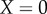	2	10	zeroth order
*β*^b^_X_	repressed production rate of gene X when the number of bound 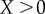	2	0	zeroth order
*β*^f^_Y_	basal production rate of gene Y when the number of bound *X* = 0	0	2	zeroth order
*β*^b^_Y_	activated production rate of gene Y when the number of bound *X* > 0	10	2	zeroth order
*δ*_*X*_	degradation rate of TF *X*	1	1	first order
*δ*_*Y*_	degradation rate of inhibitor *Y*	1	1	first order
*κ*_X_	the binding rate of TF *X* to an empty target promoter site on gene X	0	0.2*λ*	second order
*κ*_Y_	the binding rate of TF *X* to an empty target promoter site on gene Y	*λ*	0	second order
*θ*_X_	the dissociation rate of a TF *X* bound to promoter sites of X	0	0.4*λ*	first order
*θ*_Y_	the dissociation rate of a TF *X* bound to promoter sites of Y	0.5*λ*	0	first order
*α*	the association rate of *X* and *Y*	10	10	second order

**Figure 2.**
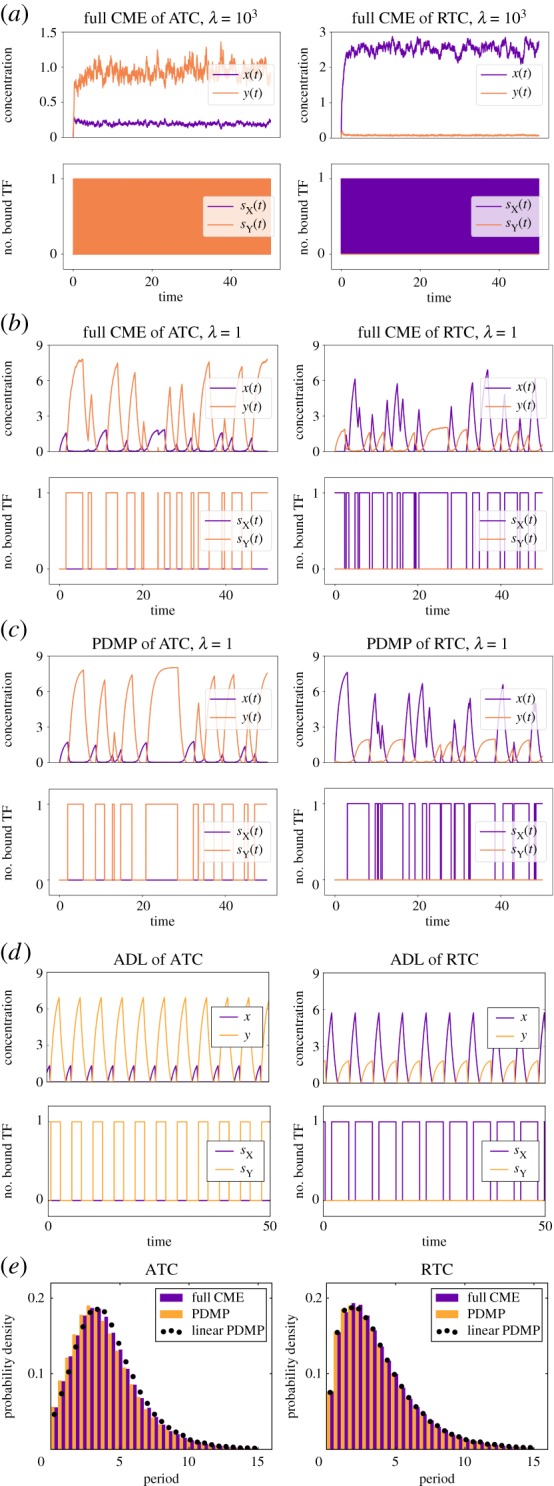
Sample paths of the full CME of the ATC and RTC in the (*a*) adiabatic regime (*λ* = 1000) and (*b*) non-adiabatic regime (*λ* = 1) for a single binding site (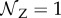). (*c*) Sample paths of the constructed piecewise deterministic Markov process when *λ* = 1 (§2.4). (*d*) The alternative deterministic limit of the processes (§2.8). (*e*) Quantification of the periods of the stochastic cycles. (Online version in colour.)

In figure 2*a*,*b*, we present sample paths of the full CME. We do not observe limit cycles in (*x*, *y*) for the idealized ATC or RTC in the fast binding and unbinding limit (i.e. adiabatic regime, *λ* = 1000), as predicted by our analysis in §2.2. When we decreased the parameter *λ* to 1, the system entered a regime where the timescale of binding and unbinding between the TF and gene is comparable to other processes. In this non-adiabatic regime, figure 2*b* shows alternating high-amplitude expression of *X* and *Y* that appears oscillatory. We measured the ‘period’ of each stochastic cycle using a protocol detailed in appendix B. The measured period of stochastic cycles exhibits a unimodal distribution with a dominant frequency, as shown in figure 2*e*.

### 2.4. Derivation of the PDMP approximating gene expression dynamics in the non-adiabatic regime

In this section, we develop the PDMP framework [21,22] of the full CME to analyse and understand the observed stochastic cycling in the non-adiabatic regime. The idea of PDMP is to reformulate the master equation conditioning on the discrete promoter states, 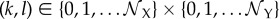. Then, for any fixed promoter states (*k*, *l*), we approximate the stochastic dynamics in the TF population space using a set of ordinary differential equations (ODEs), thus leaving the discrete and Markovian stochastic switching in the (*k*, *l*)-space. This approximation is accurate for large system size (*Ω*) or the thermodynamic limit [23,24]. The PDMP framework makes no assumptions regarding relative timescales and is equally valid for adiabatic and non-adiabatic regimes in the thermodynamic limit. To derive the PDMP, we first defined a continuum-limit probability density *p*_*k*,*l*_(*x*, *y*, *t*) ∝ *P*_*i*,*j*,*k*,*l*_(*t*) with the scaled variables *x*: = *i*/*Ω* and *y*: = *j*/*Ω*. After inserting the *p*_*k*,*l*_(*x*, *y*, *t*) into the master equation (2.7), performing a Kramers–Moyal expansion [16,17] with respect to large system size *Ω* and collecting terms to the lowest order (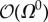), we arrived at the coupled partial differential equations for the probability density *p*_*k*,*l*_ ≡ *p*_*k*,*l*_(*x*, *y*, *t*):
2.8
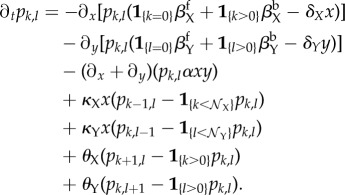


**Figure 3.**
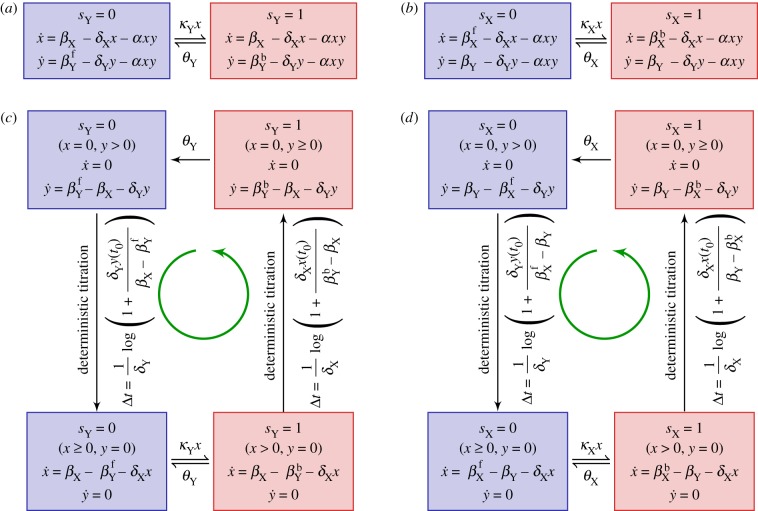
Schematic diagrams of the derived piecewise deterministic Markov process (PDMP) for (*a*) idealized activator-titration circuit (ATC) and (*b*) idealized repressor-titration circuit (RTC). Both models have a single promoter site 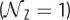. The linearized PDMP for ATC and RTC are shown in (*c*) and (*d*), respectively, where the green circular arrows indicates the direction of the emergent stochastic cycles. Dark blue 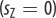 and light red 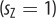 boxes denote promoter states with different production rates where 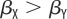 and 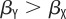, respectively. The transitions between the two regimes 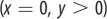 and (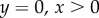) are due to titration. (Online version in colour.)

The coupled partial differential equations describe the evolution of joint probability density *p*_*k*,*l*_(*x*, *y*, *t*). Again, the ‘boundary conditions’ in the (*k*, *l*)-space, *p*_*k*,*l*_ = 0 if *k* < 0, *l* < 0, 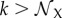 or 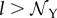, are imposed. Note that the evolution contains two parts: some terms contain ∂_*x*_ or ∂_*y*_ and describe the Liouvillian flow, whereas other terms contain *κ*_Z_ or *θ*_Z_ and describe the Markovian switching between discrete promoter states (*k*, *l*). Because the total state follows the deterministic Liouvillian flow between stochastically switching discrete state (*k*, *l*), the resulting process is referred to as the PDMP [25,26]. Equation (2.8) describes the evolution of the joint probability density. Equivalently, the sample paths of the PDMP can be described by a set of ODEs with randomly switching parameters (which is detailed in appendix D). Using the sample-path representation, the PDMP of the ATC and RTC models with a single promoter site (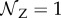) are summarized in the schematic diagrams presented in figure 3*a*,*b*.

Kinetic Monte Carlo simulations using the algorithm described in appendix C were implemented to generate the sample paths of the PDMP in the non-adiabatic (*λ* = 1) regime (figure 2*c*). These PDMP sample paths capture the salient features of the dynamics of the full CME in figure 2*b*. For example, the measured distribution of stochastic cycle periods using the PDMP is in perfect agreement with that of the full CME (figure 2*e*). Numerically, the advantage of the PDMP framework is that the kinetic Monte Carlo simulations are faster than the continuous time Markov chain simulations of the full CME because *x*, *y* are determined by numerically integrating ODEs, when the system size *Ω* ≫ 1.

### 2.5. Linearization of the PDMP

While the PDMP can be numerically simulated for any given state, the evolution of the TF concentrations is described by a set of nonlinear ODEs that do not allow for analytic solutions. The nonlinearity comes from the term *αxy*, which describes the titration (second-order reaction). We developed a linearization approximation, which uses the fast titration limit (i.e. large *αxy* compared to any other reactions) to reduce the nonlinear ODEs into linear ones. A consequence of this linearization is that each PDMP state is described by two regimes (either *x* > 0, *y* = 0 or *x* = 0, *y* > 0). In the ‘linearized PDMP’, the ODEs are linear (represented in figure 3*c*,*d*), and the transition between these two regimes is determined by determinstic titration of *x* or *y*. In each of the compartments, the linear ODE's allow analytic solutions and facilitate quantification of the random switching times. We used kinetic Monte Carlo simulations with the algorithm described in appendix C to generate sample paths of the linear PDMP in the non-adiabatic (*λ* = 1) regime. The measured distribution of stochastic cycle periods using the linear PDMP is similar to that of the full CME (figure 2*e*), although it tended to underestimate the shorter cycles that occur in the PDMP and full CME.

### 2.6. Origin of stochastic cycles

The PDMP schematic in figure 3 suggests that the stochastic cycles arise from the two-state nature of the regulated promoter, which must follow cyclical Markovian dynamics (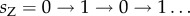). To demonstrate, we consider a two-state promoter with *constant* transition rates (*k*_+_ and *k*_−_), e.g. a promoter with a single binding site and a fixed concentration of a regulating TF. This trivial two-state promoter system generates stochastic cycles with a unimodal distribution of ‘period’ (*τ*) given by a hypoexponential distribution:
2.9
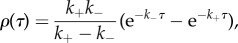
with a mean period *μ*_*τ*_ = 1/*k*_+_ + 1/*k*_−_ and variance *σ*^2^_*τ*_ = 1/*k*_+_^2^ + 1/*k*_−_^2^. The mean and variance of the period are a sum of the mean and variance of the individual transitions in the two-state cycle because the waiting times are independent. The period distribution is qualitatively similar to figure 2*e*, which suggests that stochastic dynamics of a two-state promoter explain much of the stochastic cycling observed in the single binding site ATC and RTC model. In the non-adiabatic regime, the faster protein dynamics faithfully track the underlying promoter state dynamics and generate large-amplitude stochastic cycles in (*x*, *y*).

**Figure 4.**
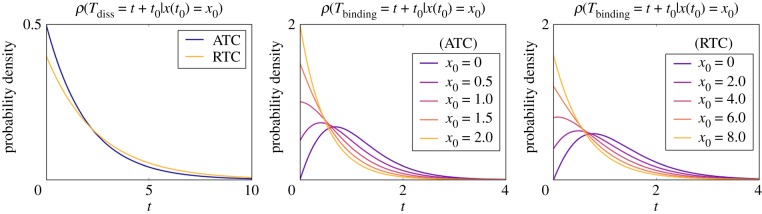
The waiting time distributions of the next dissociation and binding event in the linear PDMP. The waiting time distribution of the dissociation event is exponential. The waiting time distribution of the binding event is derived from the survival function (2.11), and depends on the initial concentration of *x*. (Online version in colour.)

This raises the question of whether stochastic cycling between two promoter states can be called oscillation. This is difficult to answer because the distinction between stochastic cycling and oscillation is ill-defined. For example, by including mechanisms that reduce variance in the timing of individual transitions, one can produce cycles that are more coherent. In the extreme limit where each transition has no variance, the period of the two-state cycle has no variance and is indistinguishable from a deterministic limit cycle. In the following section, we will investigate potential mechanisms that reduce the variance of the stochastic transition times and make the stochastic cycles more ‘deterministic’.

### 2.7. Increased coherence of stochastic cycles in ATC and RTC

The linearized ATC and RTC in figure 3*c*,*d* shows that the system often cycles through four discrete states that alternate between stochastic promoter switching and deterministic titration of *x*, *y*. The only state where the system has more than one ‘option’ is *s*_Z_ = 1 and *x* > 0 (bottom right box): it can transit to either *s*_Z_ = 0 and *x* > 0 by a dissociation event of bound *X* or to *s*_Z_ = 1 and *x* = 0 by deterministic titration of *x*. When *θ*_Z_ is sufficiently small (i.e. slow dissociation rate in the non-adiabatic regime), the system favours the latter route, which induces a ‘full cycle’ through all four discrete states in the anticlockwise order (green arrow in figure 3). As described below, this ‘full cycle’ and the *x*-dependence of the association rate conspire to reduce variance and produce more coherent stochastic cycles.

The predominant resource of uncertainty in the ‘full cycle’ of the ATC and RTC is the stochastic promoter switching (horizontal transitions) because the titration of *x* (upward arrow) and *y* (downward arrow) are deterministic and exhibit little variance. As before, the transition rate from 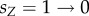 is constant and, thus, the waiting time for dissociation is a simple exponential where *ρ*(*t*) = *θ*e^−*θt*^ (figure 4*a*). Unlike the previous model, the transition from 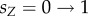 is not constant and depends on *x*(*t*), which can be quantified by computing the survival function [27]. Using the linearized ATC, *x*(*t*) can be exactly solved for the *s*_Z_ = 0 state:
2.10
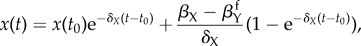
and the survival probability starting with *t* = *t*_0_ is equal to
2.11
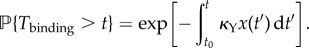
The distribution of binding times is uniquely determined by this survival function, which we plot for different initial conditions *x*_0_ in figure 4*b*. A similar calculation can be performed for the linearized RTC (figure 4*c*). The variance in binding time is reduced when initial *x*_0_ is close to zero because the system must wait until the population of *x* increases to a value above which binding is likely to take place. This explains why the ‘full cycle’ reduces variance of the total period because the system always starts at *s*_Z_ = 0 and *x* = 0 (top left box in figure 3*c*,*d*) due to the previous titration and dissociation of *x*. Thus, *x*_0_ = 0 and the waiting time before *x* binds the promoter will have reduced variance. We remark that the binding times are not exponentially distributed and are dependent on the concentration of the activator (*x*) in general. Hence, the transition is not Markovian as some of the ‘memory’ is stored in the TF space (*x*, *y*).

### 2.8. Alternative deterministic limit without invoking the adiabatic approximation

The deterministic dynamics in equation (2.5) describe the mean (*x*, *y*) concentrations in the adiabatic limit where the effective protein synthesis rates are determined by the stationary distribution of promoter states. The PDMP framework explicitly models the stochastic binding and unbinding events and is valid in both the adiabatic and non-adiabatic limits. Here, we consider an alternative ‘deterministic limit’ (ADL) of the linear PDMP, where the remaining variability due to stochastic binding and unbinding is artificially set to zero and the stochastic cycle becomes a ‘deterministic’ limit cycle. We use the first moments of the random waiting times as a deterministic residence time of a promoter state and, thus, the dynamics in (*x*, *y*) will be deterministic. The first moments can be easily computed numerically from equation (2.11) for the linear PDMP:
2.12
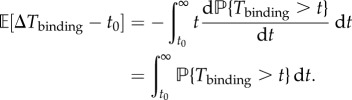


When there is more than one possible reaction, we choose the reaction with the minimal deterministic waiting time. This is analogous to Gillespie's ‘first reaction’ method [19]. For the parameter set of the idealized ATC and RTC in figure 2, the average time to titrate all the *X* is shorter than the average dissociation time and the system cycles through all four states. The time series of the ATC and RTC in the ADL is shown in figure 2*d*.

## 3. Analyses of more detailed mechanistic models

The PDMP framework will now be applied to more sophisticated models of the ATC. A previous model of the ATC [15], which we call the KB model, showed that multiple binding sites lengthened the period and improved coherence of stochastic cycling. Using the PDMP, we will show that multiple binding sites *per se* are insufficient to improve coherence. Rather, slow mRNA dynamics and multiple binding sites conspire to push the dynamics across all the promoter states and improve the coherence of stochastic cycling. The second model of the ATC [28], which we call the VKBL model, has an additional positive feedback loop where the activator activates itself in addition to activating the inhibitor. The authors previously showed that the VKBL model exhibits excitation–relaxation or noise-induced oscillation beyond the Hopf bifurcation. We will use PDMP analysis to reveal that the fluctuations in the random unbinding events of the bound TF on the promoter sites are essential for inducing transcriptional noise, which in turn drives the excitable system away from its stable fixed point and induces large excursions in a semi-periodic manner.

### 3.1. Multiple binding sites do not improve coherence of stochastic cycling in idealized ATC and RTC

**Figure 5.**
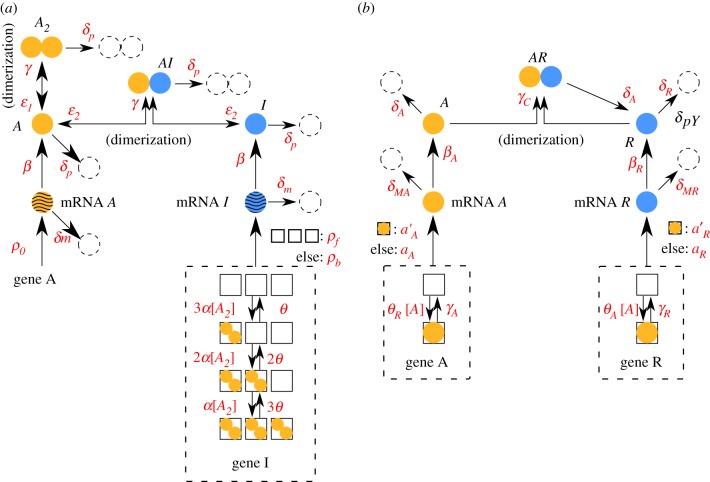
Schematic diagrams of the KB model [15] and the VKBL model [28] of the activator-titration circuit (ATC). The parametrizations were adopted from the original papers. (Online version in colour.)

**Figure 6.**
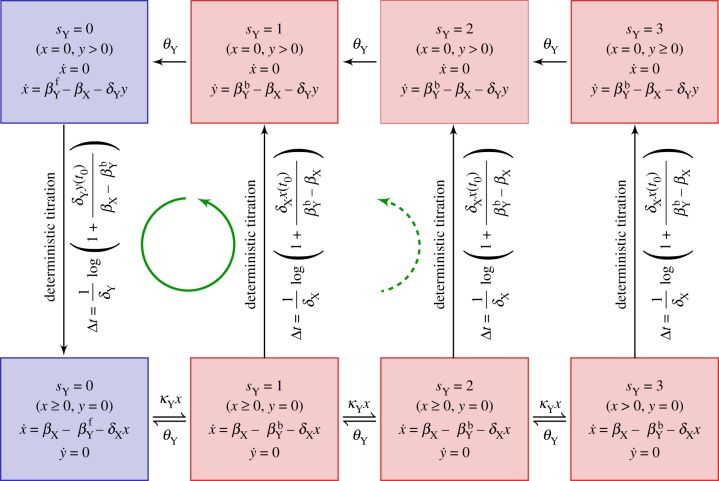
Schematic diagram of the linearized PDMP describing the idealized ATC with multiple binding sites (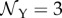) in the non-adiabatic regime. The green (circular) arrows indicates the emergent cycles which are predominantly observed in the simulations. The path of the dotted arrow is also observed, but less frequently (figure 11*b*). (Online version in colour.)

**Figure 7.**
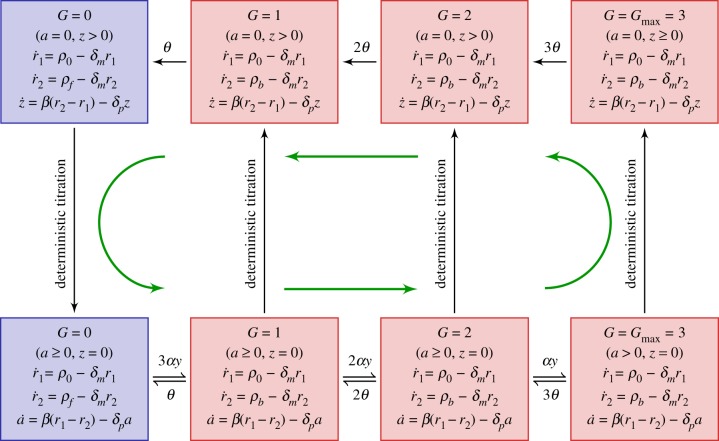
The linearized PDMP for the KB model [15]. To make the expressions compact, we define 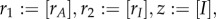 and 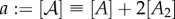. Green (circular) arrows indicate the direction of the emergent cycle which are frequently observed. (Online version in colour.)

**Figure 8.**
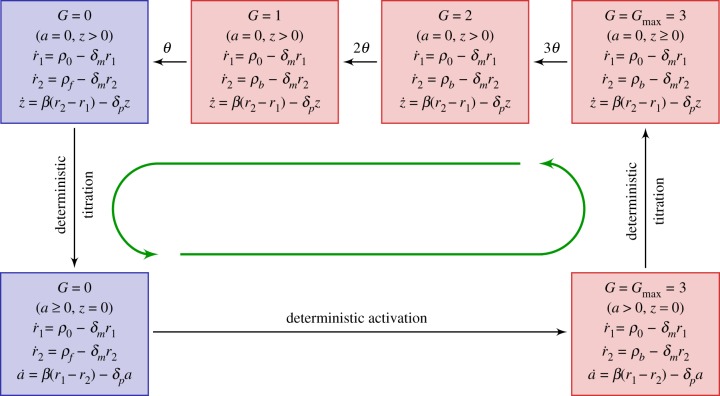
The reduced PDMP approximating the KB model [15]. The waiting time of the deterministic activation is computed as the first moment of the cumulative distribution equation (E 2). (Online version in colour.)

**Figure 9.**
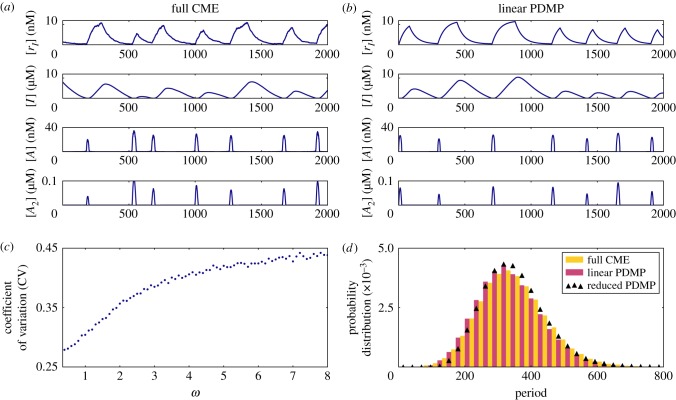
Numerically measured data of the KB model [15]. (*a*) A sample path of the full CME, (*b*) a sample path of the linear PDMP (figure 7). The stochastic cycles of the model were measured using the protocol provided in appendix B. The full CME exhibited a minor fraction of short period cycles in the genetic states. To quantify the predominant longer periods (100 ≲ period), we discarded any periods less than 50 to generate panels (*c*,*d*). Panel (*c*) presents the coefficient of variation (CV) of the stochastic periods measured in the full CME as a function of the scaling factor *ϖ*. Each point was computed from 10^4^ stochastic cycles. The larger the *ϖ*, the more short-lived is the mRNA. The long-lived mRNA introduces a delay in protein production and improves the CV. Panel (*d*) presents the probability distribution of the stochastic periods when the scaling factor *ϖ* = 1, as measured from 10^5^ stochastic cycles of the full CME, PDMP and reduced PDMP (figure 8). (Online version in colour.)

**Figure 10.**
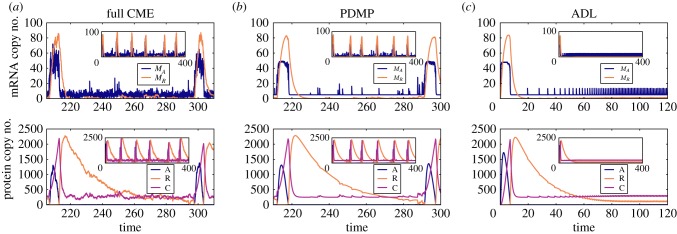
Slow fluctuations in the random binding and unbinding events induce the excitable mode of the VKBL model [28]. Column (*a*) is the full CME, (*b*) is the PDMP and (*c*) is the ADL of the VKBL model. The first row are the dynamics of the mRNAs and the second are the dynamics of the activator (A), inhibitor (R) and heterodimer complex (C). Insets are longer time series. (Online version in colour.)

**Figure 11.**
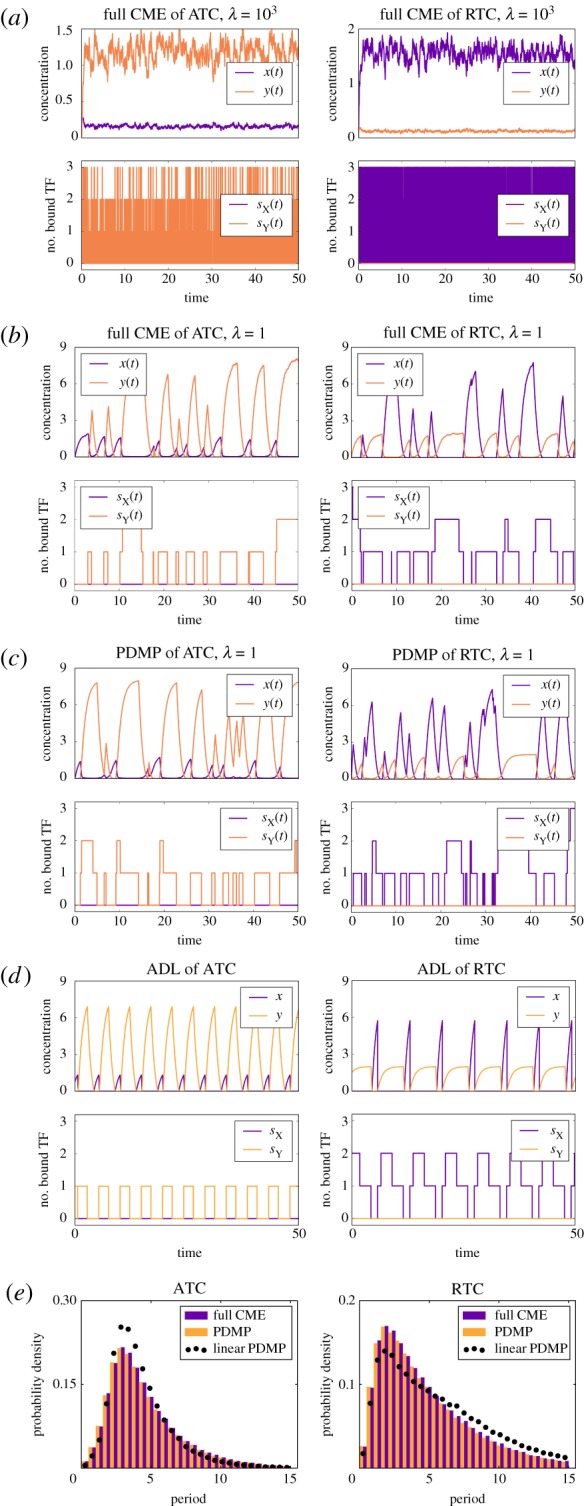
Sample paths of the full CME model of the ATC and RTC in the (*a*) adiabatic regime (*λ* = 1000) and (*b*) non-adiabatic regime (*λ* = 1) for multiple binding sites (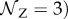. (*c*) Sample paths of the constructed piecewise deterministic Markov process when *λ* = 1 (§2.4). (*d*) The alternative deterministic limit of the processes (§2.8). (*e*) Quantification of the periods of the stochastic cycles. (Online version in colour.)

One explanation for the improved coherence of stochastic cycling in the KB model is that the coefficient of variance (CV) of the total period is reduced by increasing the number of steps in the ‘full cycle’. For example, if there are *N* independent stochastic steps in the full cycle and if the means and variances at each step are of equal magnitude, then the mean and variance of the total period scales with *N* but the CV decreases as 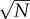. To test this idea, we simulated the idealized ATC and RTC with multiple promoter sites (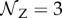) using the full CME with the parameters listed in table 1. As before, we only see stochastic cycling in the non-adiabatic limit (figure 11*a*,*b*). Strikingly, the distribution of periods was similar to that of simulations for single binding sites; compare figures 2*e*–11*e*. To understand why multiple binding sites did not increase the period or improve the coherence of stochastic cycles, we first transformed the full CME into a PDMP (appendix D). We confirmed that simulations of the PMDP accurately reproduced the results of the full CME; see figure 11*c*. We then reduced the PDMP into a linearized PDMP framework, which explains why multiple binding sites in the idealized ATC and RTC do not significantly alter the length of the period or improve coherence. The linear PDMP shows that the system becomes trapped in a ‘mini-cycle’ between the *s*_*Z*_ = 0 and *s*_*Z*_ = 1 promoter states at the blue and red boundaries (figure 6). The production rate changes instantaneously upon promoter state switching across the boundary, and deterministic titration of *x* will immediately start pushing the system upwards (red box). The timescale of titration is typically faster than that of the next stochastic binding and, thus, produces a stochastic mini-cycle around the boundary. This mini-cycle dynamic is also reflected in the ADL of the ATC and RTC, as shown in figure 11*d*.

### 3.2. Origins of improved coherence in the KB model

The KB model has several additional features compared to the idealized ATC, which could explain the observed increase in the period and coherence of stochastic cycles. First, the dynamics of mRNA transcription, degradation and protein translation are explicitly modelled. Second, the activators form homodimers before they can bind to the promoter sites and regulate gene expression. Third, the homodimers bind to the promoter sites independently and no longer need to bind sequentially (i.e. distributive binding). Last, the activator and inhibitor heterodimer is no longer irreversible and can dissociate to form monomers.

Below, we describe PDMP analysis of the KB model [15] for the ATC shown in figure 5*a*. Beginning with the master equation governing the KB model, we performed the system-size expansion presented in §2.4 and arrived at the following PDMP:
3.1
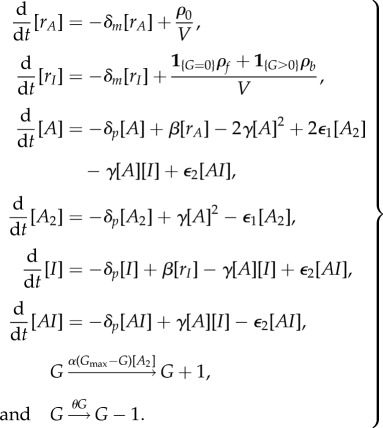
We used the same variables and parameter set in figure 6 of the original paper [15]. The state variables, [*r*_*A*_], [*r*_*I*_], [*A*], [*A*_2_], [*I*], [*AI*] and *G* are the concentrations of the activator mRNA, inhibitor mRNA, monomeric activators, homodimeric activators, inhibitors and heterodimers. *G* is the promoter state variable, 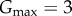 is the total number of binding sites and *V* is analogous to the system size. The association and dissociation rates are multiplied by *G* and (*G*_max_ − *G*) because there are multiple combinations of promoters with the same number of bound activators due to distributive binding. We reduced the PDMP into a linear PDMP (appendix E). The Monte Carlo kinetic simulation of the linear PDMP gives similar results to the full CME of the KB model; see figure 9*a*,*b*. In both cases, the stochastic cycles exhibit a well-defined distribution of periods with reduced CV, as previously observed.

The schematic of the linear PDMP in figure 7 suggests that the period and coherence improved because the mRNA dynamics introduce a time lag between the change in mRNA production rate and the resulting protein synthesis rate. Thus, even though the transcription rate changes instantaneously upon crossing the boundary when 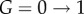, the mRNA levels will respond and reach a new state on the timescale set by the mRNA degradation rate *δ*_*m*_. This lag delays the process of deterministic titration, which requires new inhibitor synthesis, such that *G* can reach saturation before *x* is titrated. As a consequence, the KB model now goes through the largest cycle from *G* = 0 to *G* = 3. Given the importance of the lag, we expect the coherence of stochastic cycling to decrease upon increasing the rate of mRNA degradation and, thus, making the mRNA more responsive to changes in transcription. We tested this idea by rescaling the mRNA degradation 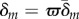 and protein translation 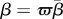, such that the total protein levels stayed fixed, but the mRNA degradation rate could be varied through *ϖ*. Our results in figure 9*c* confirm that increasing the mRNA degradation rate via larger *ϖ* created shorter and less coherent stochastic ‘mini-cycles’, similar to the idealized ATC which had a measured CV = 0.622.

We noted that the variance of the waiting times of binding events in the linear PDMP was much less than those of unbinding events. This motivated us to keep only the stochastic unbinding events whose waiting times are all exponentially distributed and take a deterministic waiting time for the binding events by evaluating the first moment of the cumulative distribution (E 2). The resulting model is summarized in figure 8 and is referred to as the reduced PDMP. In the reduced model, the only stochasticity—the random unbinding events—results in a random duration in a series of promoter states which actively produce the inhibitor *I* (top row of figure 8). The excellent agreement between the reduced PDMP and full CME of the KB model suggests that the variability in stochastic cycle times is mostly determined by the stochasticity of unbinding events (figure 9*d*). Although the serial nature of unbinding events helps reduce the overall CV and improve the coherence of stochastic cycling, the randomness of unbinding events propagates nonlinearly and contributes to the overall uncertainty of the stochastic cycle period. For example, in each ‘episode’ of serial stochastic unbinding, the number of synthesized inhibitors will be a random quantity that subsequently determines the time to titrate the produced *I* back to zero (downward arrow) before the promoter state can deterministically cycle back to the actively producing *I* state (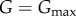 and *a* = 0).

### 3.3. Noise-induced oscillation in the VKBL model

We then turned our attention to the ATC model studied by Vilar *et al.* [28], whose schematic is shown in figure 5*b*. In the VKBL model, the activator activates itself in addition to the inhibitor and, thus, can exhibit deterministic limit cycles. However, the authors deliberately studied the VKBL model for a parameter set where there were no deterministic limit cycles but the system exhibited excitation–relaxation or noise-induced oscillations. Below, we will use PDMP analysis to show that stochastic promoter fluctuations are responsible for kicking the stable fixed point into an excitable excursion. The PDMP of the VKBL model is given by
3.2
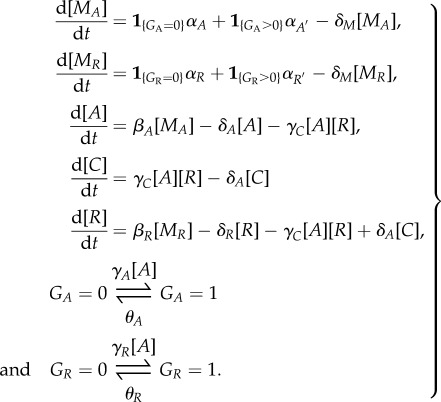
We adopt the same symbols and parameters of figure 5 from the original work [28], except for discrete *G*_A_ ∈ {0, 1} and *G*_R_ ∈ {0, 1}, which represent the number of bound activators on the promoters of A or R.

The sample path of the PDMP faithfully captures the signature of the dynamics of the full CME in the parameter regime with noise-induced oscillations; cf. figure 10*a*,*b*. The PDMP only takes into account the stochasticity of the binding and unbinding events (i.e. 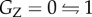); the rest of the processes are described by deterministic evolutionary equations. Thus, we can conclude that the noise-induced oscillations in the full CME are due to the discrete binding and unbinding events at the promoter site. In both the full CME and PDMP, the system constantly switches back and forth between 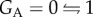 and produces a bursty activator mRNA population (figure 10*a*,*b*). However, occasionally, an unbinding event takes longer than usual, which leads to a larger-than-average number of activator mRNAs. This larger-than-average number of activators titrates all the inhibitors (*R*) and the critical accumulation of activator excites the system through a large excursion in the phase space; see figure 10*b*. The ADL of the VKBL model does not exhibit any excitable excursions, as shown in figure 10*c*. By definition, the ADL does not exhibit any variability in the binding and unbinding events. The lack of excitable excursions in the ADL is consistent with the idea that rare fluctuations in the unbinding times are the critical ingredient for generating enough activators (*A*) to titrate all the inhibitors (*R*) in the system.

## 4. Discussion and future outlook

Dynamical models of gene expression often assume that switching between promoter states (e.g. binding and unbinding of regulatory proteins) takes place at a much shorter timescale than any other processes in the model. This idealization, known as the quasi-steady-state or adiabatic approximation [2,3], uses an effective rate inferred from the QSD of the promoter states. When the timescale of promoter state switching is comparable to other processes, which is typically the case for natural systems, this approach fails to describe the resulting dynamics accurately.

In this article, we investigated the stochastic dynamics of biological clocks in the non-adiabatic regime. Previous work [14,15] demonstrated that time delays, which arise from slow promoter switching in the non-adiabatic regime, are important for the emergence of deterministic limit cycles. These studies modelled the transcription rate as an ensemble-averaged transcription rate of the discrete promoter states. Such a treatment would be precise if one had a large copy number of independent promoters, e.g. models in [29]. However, the copy number of genomic DNA is small in many biological systems and the transcription rate at any given time can only be one of the two discrete values *β*^b^_Z_ and *β*^f^_Z_. When the switching timescale is fast (i.e., adiabatic), the promoter state goes through a large number of cycles between consecutive transcription events, and the effective rate of transcription converges to the ensemble-averaged transcription rate. However, when the switching timescale is slow (i.e. non-adiabatic), the averaged transcription rate cannot capture the nature of alternating rates of transcription events.

The effects of non-adiabatic promoter fluctuations have been investigated, mostly numerically, in the literature of biological clocks. Both the studies of Potoyan & Wolynes [30] and Gonze *et al.* [31] reported that slower switching rates compromise the coherent oscillation seen in the deterministic limit. In a slightly different model, Feng *et al.* [32] observed coherent oscillation when the binding and unbinding events were either very fast (adiabatic) or very slow (non-adiabatic). Last, stochastic resonance was reported by Li & Li [33], who showed that there exists a ‘sweet spot’ where the promoter switching is neither fast or slow. In the above-mentioned studies, the adopted methods range from direct computation of the eigenvalues of the truncated CME [30], direct computation of the stationary distribution [32], and direct continuous-time Markov simulations and power spectral analyses of the generated sample paths [31,33]. Although it is straightforward to carry out these analyses, they reveal little about the mechanisms of stochastic oscillations. For example, these methodologies could not answer why a system with more promoter binding sites exhibits more coherent oscillation, or quantify the impact of mRNA or post-translational reactions, e.g. dimerization.

We present a mathematical framework to analyse the stochastic dynamics of gene expression in the non-adiabatic regime. In this framework, we begin with the most detailed description of the individual molecular-based and stochastic dynamics, the CME and systematically construct the PDMPs, which retains the discrete and stochastic switching nature of the genetic states. This framework is a natural generalization of our previous work [11,21,22], and the derived PDMP has been shown to be a powerful mathematical tool to model coloured noise in stochastic gene expression [12,34–42]. Our analyses showed that, for the models we investigated, the PDMP faithfully captures dynamical features of the individual molecular-based models. We further proposed a scheme to construct an alternative ‘deterministic description’ of the dynamics without invoking the adiabatic assumption. These analytical tools revealed the emergent non-equilibrium transitions between the discrete genetic states in the non-adiabatic regime. In the idealized models, both the ATC and RTC exhibited stochastic cycling in the discrete genetic states. We showed that a more robust and coherent oscillation (the full cycle) occurred in a regime of slower dissociation rate (small *θ*_Z_ in the idealized model). The analysis also revealed the interactions between the TF population and the transition between the discrete genetic states, showing that it is necessary to consider the joint process describing the TF dynamics and gene switching dynamics. While the joint process (PDMP) is Markovian, it is known that the TF dynamics alone [43] is non-Markovian. In this work, we showed that the gene switching dynamics alone is also non-Markovian.

To illustrate the practicality of these analytical tools, we analysed more sophisticated and detailed models. Interestingly, in the two models we investigated, the analysis revealed different mechanisms of to induce oscillations. In the KB model [15], we found that the oscillation was induced by the slow-transitions between the discrete genetic states, similar to the idealized models. Nevertheless, in contrast to the idealized model which exhibits similar dynamics when we changed the number of promoter sites 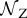, the inclusion of the mRNA in the KB model pushes the system to transit through more genetic states. This is because the product of the gene, i.e. mRNA, no longer directly (and abruptly) regulates its own production rate and there is a delay. As the predominant stochasticity of the system arises from the random unbinding events, the ability to travel through more internal stages decreases the coefficient of variance. Consequently, more coherent oscillations were observed in the KB model, compared to the idealized models. By applying the analytical tools to the VKBL model [28], we were able to show that the average (deterministic) genetic switching events were not sufficient to induce the oscillation. Instead, longer-than-average binding events were responsible for exciting the FitzHugh–Nagumo-like system to go through a large excursion in the phase space. Biologically, these longer-than-average binding events are called transcriptional bursting noise. It is straightforward to show that by increasing translational bursting, achieved by simultaneously scaling up the translation rate and scaling down the transcription rate, one can also induce similar oscillations (data not shown).

On a final note, the PDMP is derived from the detailed CME and can be viewed as a hybrid model which combines the continuous and deterministic TF dynamics and the discrete and stochastic promoter switching dynamics. The PDMP is related to other stochastic-hybrid approaches [44–49]. However, on a conceptual level, we explicitly demonstrate how the PDMP arises from the fully discrete CMEs in the limit of large protein numbers (e.g. eukaryotic cells). We further show that the PDMP accurately captures stochastic gene dynamics of the full CME in the non-adiabatic regime. The PDMP is therefore a promising ‘bridge model’ connecting detailed and mechanistic computational models and highly idealized discrete-state oscillators [50–52], phase oscillators [53–56] or one-dimensional delay-induced oscillators [57,58] which were previously proposed *ad hoc*.

## Data accessibility

This article has no additional data.

## Appendix A. Scaling relationship between parameters in the mass action kinetics and parameters in the full CME model

The scaling of parameters depends on the order of the reactions. The mapping from the defined mass action rates to the rates in the full CME simulations are:

A 1a

A 1b

and

A 1c

with Z ∈ {X, Y}.

## Appendix B. Measuring the periods of stochastic cycles

In the marginal space describing the genetic state (*s*_Z_), the regulatory protein dynamic of *s*_Z_ = 0 is significantly different from the other states (*s*_Z_ > 0). This is because we defined the transcription rate of the regulated gene Z to be **1**_{s_Z___> 0}_*β*^b^_Z_ + **1**_{*s*_Z___= 0}_*β*^f^_Z_. Therefore, we record the times at which each transition  occurs, and we define the elapsed time between two consecutive transitions as the period of the stochastic cycle. We measured 10^5^ periods for each model and compared their probability distributions in figure 2*e*.

## Appendix C. A kinetic Monte Carlo scheme to generate sample paths of nonlinear PDMPs

When the deterministic part of the PDMP has an analytical solution, Bokes *et al.* prescribed a simple algorithm to generate the exact random switching times [59]. We used the Bokes algorithm for our linearized PDMP, which has an analytical solution for the deterministic part. The two-dimensional deterministic system of the full PDMP is governed by a set of nonlinear equations where the nonlinearity comes from the term describing heterodimer formation, *αxy*. As the exact solution is unknown, we used the following numerical scheme to generate exact sample paths that respect the waiting times before the next switching events. In our idealized model of the ATC or RTC, there is only one gene whose promoter states can switch. Our algorithm below generates sample paths for this specific type of network with one promoter *s*_*Z*_, but it can be generalized to more complex, multiple switching genes.

(1) *Initiation*. Initiate the state variable (*x*, *y*, *s*_*Z*_). Here (*x*, *y*) are the population density of the TFs, and *s*_*Z*_ is the discrete promoter state; in ATC, *s*_*Z*_ = *s*_Y_ and in RTC, *s*_*Z*_ = *s*_X_. Initiate a ‘time of last switching’ .
(2) *Generate dissociation time*. When *s*_*Z*_ > 0, the bound X can dissociate from the promoter sites. The exponentially distributed waiting time is generated by assigning , with *u* ∼ Unif(0, 1). When *s*_*Z*_ = 0, assign .
(3) *Generate a random number to determine the next binding event*. When *s*_*Z*_ is less than the maximum capacity of promoter sites (ATC: ; RTC: ), it is possible to have a binding event in the future. Generate a *u*_1_ ∼ Unif(0, 1) for future use. When *s* is equal to the maximum capacity of the promoter sites, we set .
(4) *Forward integrate the system*. Advance the time by a small d*t* ≪ 1 to forward integrate the ODEs numerically; update the state (*x*, *y*). We implement the integrator using the Runge–Kutta method.
(5) *Check if a binding or dissociation event occurs*. If the time *t* > *T*_diss_, there was a dissociation event that occurred in the past time step. Update the genetic state . On the other hand, the probability that the system has not bound another TF molecule is
  C 1
  We compute this quantity, noting that this can be summed up for each of the time steps d*t* numerically. If , we know by the inverse transform sampling that a binding event occurred in the past time step, so assign  accordingly.
(6) *Repeat*. If there was a change in the promoter state *s*_*Z*_, then repeat from 2 and register a new *t*_0_; otherwise, repeat from 4, until the end of the simulation.

We remark that if the dynamics are linear and solvable, one can analytically compute the survival function equation (C 1) and derive a more efficient continuous-time sampling technique [59]. In the VKBL model, the above algorithm is generalized to the two genetic states (*G*_*A*_, *G*_*R*_). We note that there are four distinct genetic states *ψ*_1_ := (0, 0), *ψ*_2_ := (1, 0), *ψ*_3_ := (0, 1) and *ψ*_4_ := (1, 1), and the possible transitions are . Therefore, when the genetic state is in *ψ*_1_, we derive two survival functions and use them to perform inverse sampling which generates a first binding event. Similarly, when the genetic state is in *ψ*_4_, two exponentially distributed dissociation times are to be sampled to determine the first dissociation event. As for genetic states *ψ*_2_ and *ψ*_3_, they can either transit to *ψ*_1_ (by a dissociating event of the bound TF) or *ψ*_4_ (by a binding event between the free promoter and a free TF). The random times are sampled similar to the above steps 2–5.

## Appendix D. PDMP of idealized ATC and RTC

Like all random processes, there are two representations for a general PDMP (linear or nonlinear; see below). The forward Chapman–Kolmogorov equation (2.8) of the joint probability density is a set of linear partial differential equations of the form

D 1

where **P** is a vector of joint probability density and  is the transition operator.

An equivalent representation of the PDMP is to describe the evolution of the sample paths. In this representation, the PDMP is a process described by ODEs with randomly switching parameters. Using this approach, the PDMP of the ATC is

D 2

and the PDMP of the RTC is

D 3

where  is the number of promoter states.

## Appendix E. Linear PDMP of the KB model

We performed the following model reduction for the nonlinear PDMP in equation (3.1) by imposing the following assumptions based on the parameters used in [15]:

### E.1. Irreversible heterodimerization

The heterodimerization is much larger than the reverse rate, and we approximate it as an irreversible process. Thus, [*AI*] is ignored.

### E.2. Fast homodimerization and dissociation

The homodimerization and dissociation occurs at a much faster timescale than other processes, so the concentrations of [*A*] and [*A*_2_] satisfy the quasi-stationary approximation—*γ*[*A*]^2^ ≈ *ε*_1_[*A*_2_]—at any given time. For simplicity, we define  as the total number of activators in monomeric and dimeric form.

### E.3. Linearization

We assume that, at any given time, either activators or inhibitors are dominant such that the other becomes a limiting factor. This is the approximation we proposed in §2.8 for the idealized model. After this linearization, the dynamics of [*r*_*A*_], [*r*_*I*_],  and [*I*] are all analytically tractable. In the long run, , and [*r*_*I*_] relax exponentially to the fixed points *ρ*_*f*_/*δ*_*m*_ and *ρ*_*b*_/*δ*_*m*_ when *G* = 0 and *G* > 0, respectively. The activation rate of the linearized PDMP is proportional to the concentration of the dimeric activators *A*_2_, which can be solved by equating

E 1

using the adiabatic approximation *γ*[*A*]^2^ = *ε*_1_[*A*_2_] in the last step. The survival function of the waiting time of the next binding event can be formulated as follows: let the random time to the next binding event be *τ*,

E 2

The survival function is then used to generate the stochastic waiting time to the next binding event using the inverse transform sampling method.

## References

[1] Buchler NE, Gerland U, Hwa T 2003 On schemes of combinatorial transcription logic. Proc. Natl Acad. Sci. USA 100, 5136–5141. (10.1073/pnas.0930314100) 12702751 PMC404558

[2] Hornos J, Schultz D, Innocentini G, Wang J, Walczak A, Onuchic J, Wolynes PG 2005 Self-regulating gene: an exact solution. Phys. Rev. E 72, 051907 (10.1103/PhysRevE.72.051907) 16383645

[3] Ackers GK, Johnson AD, Shea MA 1982 Quantitative model for gene regulation by lambda phage repressor. Proc. Natl Acad. Sci. USA 79, 1129–1133. (10.1073/pnas.79.4.1129) 6461856 PMC345914

[4] Kwon H, Park S, Lee S, Lee DK, Yang CH 2001 Determination of binding constant of transcription factor AP-1 and DNA. FEBS. J. 268, 565–572. (10.1046/j.1432-1327.2001.01897.x) 11168395

[5] Kyo M, Yamamoto T, Motohashi H, Kamiya T, Kuroita T, Tanaka T, Engel JD, Kawakami B, Yamamoto M 2004 Evaluation of MafG interaction with Maf recognition element arrays by surface plasmon resonance imaging technique. Genes Cells 9, 153–164. (10.1111/j.1356-9597.2004.00711.x) 15009092

[6] Geertz M, Shore D, Maerkl SJ 2012 Massively parallel measurements of molecular interaction kinetics on a microfluidic platform. Proc. Natl Acad. Sci. USA 109, 16 540–16 545. (10.1073/pnas.1206011109) PMC3478601 23012409

[7] Hammar P, Walldén M, Fange D, Persson F, Baltekin Ö, Ullman G, Leroy P, Elf J 2014 Direct measurement of transcription factor dissociation excludes a simple operator occupancy model for gene regulation. Nat. Genet. 46, 405–408. (10.1038/ng.2905) 24562187 PMC6193529

[8] Walczak AM, Mugler A, Wiggins CH 2012 Analytic methods for modeling stochastic regulatory networks. In *Computational modeling of signaling networks*. *Methods in Molecular Biology* (*Methods and Protocols*), vol. 880 (eds Liu X, Betterton M). Totowa, NJ: Humana Press (10.1007/978-1-61779-833-7_13)

[9] Bressloff PC 2017 Stochastic switching in biology: from genotype to phenotype. J. Phys. A: Math. Theor. 50, 133001 (10.1088/1751-8121/aa5db4)

[10] Thomas P, Popović N, Grima R 2014 Phenotypic switching in gene regulatory networks. Proc. Natl Acad. Sci. USA 111, 6994–6999. (10.1073/pnas.1400049111) 24782538 PMC4024914

[11] Hufton PG, Lin YT, Galla T, McKane AJ 2016 Intrinsic noise in systems with switching environments. Phys. Rev. E 93, 052119 (10.1103/PhysRevE.93.052119) 27300842

[12] Jia C 2017 Simplification of Markov chains with infinite state space and the mathematical theory of random gene expression bursts. Phys. Rev. E 96, 032402 (10.1103/PhysRevE.96.032402) 29346865

[13] Al-Radhawi MA, Del Vecchio D, Sontag ED 2017 Multi-modality in gene regulatory networks with slow gene binding. arXiv,1705.02330. (http://arxiv.org/abs/1705.02330)

[14] François P, Hakim V 2005 Core genetic module: the mixed feedback loop. Phys. Rev. E 72, 031908 (10.1103/PhysRevE.72.031908) 16241483

[15] Karapetyan S, Buchler NE 2015 Role of DNA binding sites and slow unbinding kinetics in titration-based oscillators. Phys. Rev. E 92, 062712 (10.1103/PhysRevE.92.062712) PMC4777296 26764732

[16] van Kampen NG 2007 Stochastic processes in physics and chemistry. Amsterdam, The Netherlands: North-Holland.

[17] Gardiner CW 2004 Handbook of stochastic methods. Berlin, Germany: Springer-Verlag.

[18] Khalil HK 1996 Nonlinear systems. Englewood Cliffs, New Jersey: Prentice-Hall.

[19] Gillespie DT 1976 A general method for numerically simulating the stochastic time evolution of coupled chemical reactions. J. Comput. Phys. 22, 403–434. (10.1016/0021-9991(76)90041-3)

[20] Gillespie DT 1977 Exact stochastic simulation of coupled chemical reactions. J. Phys. Chem. 81, 2340–2361. (10.1021/j100540a008)

[21] Lin YT, Doering CR 2016 Gene expression dynamics with stochastic bursts: construction and exact results for a coarse-grained model. Phys. Rev. E 93, 022409 (10.1103/PhysRevE.93.022409) 26986364

[22] Lin YT, Galla T 2016 Bursting noise in gene expression dynamics: linking microscopic and mesoscopic models. J. R. Soc. Interface 13, 20150772 (10.1098/rsif.2015.0772) 26763330 PMC4759790

[23] Kurtz TG 1970 Solutions of ordinary differential equations as limits of pure jump Markov processes. J. Appl. Probab. 7, 49–58. (10.2307/3212147)

[24] Jahnke T, Kreim M 2012 Error bound for piecewise deterministic processes modeling stochastic reaction systems. Multiscale Model. Simul. 10, 1119–1147. (10.1137/120871894)

[25] Davis MH 1984 Piecewise-deterministic Markov processes: a general class of non-diffusion stochastic models. J. R. Stat. Soc. Ser. B 46, 353–388.

[26] Faggionato A, Gabrielli D, Crivellari MR 2009 Non-equilibrium thermodynamics of piecewise deterministic Markov processes. J. Stat. Phys. 137, 259–304. (10.1007/s10955-009-9850-x)

[27] Cox DR, Oakes D 1984 Analysis of survival data, vol. 21 Boca Raton, FL: CRC Press.

[28] Vilar JM, Kueh HY, Barkai N, Leibler S 2002 Mechanisms of noise-resistance in genetic oscillators. Proc. Natl Acad. Sci. USA 99, 5988–5992. (10.1073/pnas.092133899) 11972055 PMC122889

[29] Wang Z, Potoyan DA, Wolynes PG 2016 Molecular stripping, targets and decoys as modulators of oscillations in the NF-*κ*B/I*κ*B*α*/DNA genetic network. J. R. Soc. Interface 13, 20160606 (10.1098/rsif.2016.0606) 27683001 PMC5046959

[30] Potoyan DA, Wolynes PG 2014 On the dephasing of genetic oscillators. Proc. Natl Acad. Sci. USA 111, 2391–2396. (10.1073/pnas.1323433111) 24469814 PMC3926054

[31] Gonze D, Halloy J, Goldbeter A 2004 Emergence of coherent oscillations in stochastic models for circadian rhythms. Phys. A: Stat. Mech. Appl. 342, 221–233. (10.1016/j.physa.2004.04.082)

[32] Feng H, Han B, Wang J 2012 Landscape and global stability of nonadiabatic and adiabatic oscillations in a gene network. Biophys. J. 102, 1001–1010. (10.1016/j.bpj.2012.02.002) 22404922 PMC3296035

[33] Li D, Li C 2008 Noise-induced dynamics in the mixed-feedback-loop network motif. Phys. Rev. E 77, 011903 (10.1103/PhysRevE.77.011903) 18351872

[34] Friedman N, Cai L, Xie XS 2006 Linking stochastic dynamics to population distribution: an analytical framework of gene expression. Phys. Rev. Lett. 97, 168302 (10.1103/PhysRevLett.97.168302) 17155441

[35] Lipniacki T, Paszek P, Marciniak-Czochra A, Brasier AR, Kimmel M 2006 Transcriptional stochasticity in gene expression. J. Theor. Biol. 238, 348–367. (10.1016/j.jtbi.2005.05.032) 16039671

[36] Ge H, Qian H, Xie XS 2015 Stochastic phenotype transition of a single cell in an intermediate region of gene state switching. Phys. Rev. Lett. 114, 078101 (10.1103/PhysRevLett.114.078101) 25763973

[37] Li Y, Xu Y, Kurths J, Yue X 2016 Lévy-noise-induced transport in a rough triple-well potential. Phys. Rev. E 94, 042222 (10.1103/PhysRevE.94.042222) 27841518

[38] Jia C, Zhang MQ, Qian H 2017 Emergent Lévy behavior in single-cell stochastic gene expression. Phys. Rev. E 96, 040402 (10.1103/PhysRevE.96.040402) 29347590

[39] Bokes P, Singh A 2017 Gene expression noise is affected differentially by feedback in burst frequency and burst size. J. Math. Biol. 74, 1483–1509. (10.1007/s00285-016-1059-4) 27665109

[40] Wang H, Cheng X, Duan J, Kurths J, Li X 2017 Likelihood for transcriptions in a genetic regulatory system under asymmetric stable Lévy noise. arXiv,1705.06479. (http://arxiv.org/abs/1705.06479)

[41] Bokes P, Singh A, Lin YT 2017 High cooperativity in negative feedback can amplify noisy gene expression, p. 125914. bioRxiv (10.1101/125914)

[42] Lin YT, Hufton PG, Lee EJ, Potoyan DA 2017 A stochastic and dynamical view of pluripotency in mouse embryonic stem cells. arXiv, 1710.08542. (http://arxiv.org/abs/1710.08542)

[43] Bena I 2006 Dichotomous Markov noise: exact results for out-of-equilibrium systems. Int. J. Mod. Phys. B 20, 2825–2888. (10.1142/S0217979206034881)

[44] Newby JM, Bressloff PC, Keener JP 2013 Breakdown of fast-slow analysis in an excitable system with channel noise. Phys. Rev. Lett. 111, 128101 (10.1103/PhysRevLett.111.128101) 24093303

[45] Bressloff PC, Newby JM 2014 Stochastic hybrid model of spontaneous dendritic NMDA spikes. Phys. Biol. 11, 016006 (10.1088/1478-3975/11/1/016006) 24476677

[46] Bressloff PC, Bressloff BPC, Bressloff PC 2015 Path-integral methods for analyzing the effects of fluctuations in stochastic hybrid neural networks. J. Math. Neurosci. 5, 4 (10.1186/s13408-014-0016-z) 25852979 PMC4385107

[47] Bressloff PC, Faugeras O 2017 On the Hamiltonian structure of large deviations in stochastic hybrid systems. J. Stat. Mech. J. Stat. Mech. 2017, 033206 (10.1088/1742-5468/aa64f3)

[48] Bressloff PC, Lawley SD 2017 Mean first passage times for piecewise deterministic Markov processes and the effects of critical points. Mech. J. Stat. Mech. 2017, 063202 (10.1088/1742-5468/aa71df)

[49] Bressloff PC 2017 Feynman–Kac formula for stochastic hybrid systems. Phys. Rev. E 95, 012138 (10.1103/PhysRevE.95.012138) 28208495

[50] Pinto IL, Escaff D, Harbola U, Rosas A, Lindenberg K 2014 Globally coupled stochastic two-state oscillators: fluctuations due to finite numbers. Phys. Rev. E 89, 052143 (10.1103/PhysRevE.89.052143) 25353775

[51] Rosas A, Escaff D, Lindenberg K, Pinto IL 2016 Globally coupled stochastic two-state oscillators: synchronization of infinite and finite arrays. J. Phys. A: Math. Theor. 49, 095001 (10.1088/1751-8113/49/9/095001)

[52] Escaff D, Pinto IL, Lindenberg K 2014 Arrays of stochastic oscillators: nonlocal coupling, clustering, and wave formation. Phys. Rev. E 90, 052111 (10.1103/PhysRevE.90.052111) 25493744

[53] Schwabedal JT, Pikovsky A 2010 Effective phase description of noise-perturbed and noise-induced oscillations. Eur. Phys. J. Spec. Top. 187, 63–76. (10.1140/epjst/e2010-01271-6)

[54] Schwabedal JT, Pikovsky A 2010 Effective phase dynamics of noise-induced oscillations in excitable systems. Phys. Rev. E 81, 046218 (10.1103/PhysRevE.81.046218) 20481818

[55] Schwabedal JT, Pikovsky A 2013 Phase description of stochastic oscillations. Phys. Rev. Lett. 110, 204102 (10.1103/PhysRevLett.110.204102) 25167416

[56] Newby JM, Schwemmer MA 2014 Effects of moderate noise on a limit cycle oscillator: counterrotation and bistability. Phys. Rev. Lett. 112, 114101 (10.1103/PhysRevLett.112.114101) 24702373

[57] Mather W, Bennett MR, Hasty J, Tsimring LS 2009 Delay-induced degrade-and-fire oscillations in small genetic circuits. Phys. Rev. Lett. 102, 1–4. (10.1103/PhysRevLett.102.068105) PMC2924583 19257639

[58] Mather W, Hasty J, Tsimring LS 2014 Synchronization of degrade-and-fire oscillations via a common activator. Phys. Rev. Lett. 113, 1–5. (10.1103/PhysRevLett.113.128102) PMC4494757 25279645

[59] Bokes P, King JR, Wood AT, Loose M 2013 Transcriptional bursting diversifies the behaviour of a toggle switch: hybrid simulation of stochastic gene expression. Bull. Math. Biol. 75, 351–371. (10.1007/s11538-013-9811-z) 23354929

